# Non-Ionizing Radiation for Cardiac Human Amniotic Mesenchymal Stromal Cell Commitment: A Physical Strategy in Regenerative Medicine

**DOI:** 10.3390/ijms19082324

**Published:** 2018-08-08

**Authors:** Mario Ledda, Enrico D’Emilia, Maria Grazia Lolli, Rodolfo Marchese, Claudio De Lazzari, Antonella Lisi

**Affiliations:** 1 Institute of Translational Pharmacology, National Research Council, (CNR), via del Fosso del Cavaliere 100, 00133 Rome, Italy; mario.ledda@ift.cnr.it (M.L.); mariagrazia.lolli@ift.cnr.it (M.G.L.); 2 Dipartimento Innovazioni Tecnologiche (INAIL-DIT), Via Fontana Candida 1, Monte Porzio Catone, 00078 Rome, Italy; e.demilia@inail.it; 3 Research Center, FBF S. Peter Hospital, Via Cassia 600, 00189 Rome, Italy; marchese.rodolfo@fbfrm.it; 4 Institute of Clinical Physiology, National Research Council, (CNR), Via Palestro 32, 00185 Rome, Italy; dela@ifc.cnr.it

**Keywords:** hAMSCs, cardiac commitment, angiogenic commitment, Nitric Oxide, ELF-EMF, regenerative medicine

## Abstract

Cell therapy is an innovative strategy for tissue repair, since adult stem cells could have limited regenerative ability as in the case of myocardial damage. This leads to a local contractile dysfunction due to scar formation. For these reasons, refining strategy approaches for “in vitro” stem cell commitment, preparatory to the “in vivo” stem cell differentiation, is imperative. In this work, we isolated and characterized at molecular and cellular level, human Amniotic Mesenchymal Stromal Cells (hAMSCs) and exposed them to a physical Extremely Low Frequency Electromagnetic Field (ELF-EMF) stimulus and to a chemical Nitric Oxide treatment. Physically exposed cells showed a decrease of cell proliferation and no change in metabolic activity, cell vitality and apoptotic rate. An increase in the mRNA expression of cardiac and angiogenic differentiation markers, confirmed at the translational level, was also highlighted in exposed cells. Our data, for the first time, provide evidence that physical ELF-EMF stimulus (7 Hz, 2.5 µT), similarly to the chemical treatment, is able to trigger hAMSC cardiac commitment. More importantly, we also observed that only the physical stimulus is able to induce both types of commitments contemporarily (cardiac and angiogenic), suggesting its potential use to obtain a better regenerative response in cell-therapy protocols.

## 1. Introduction

In the area of regenerative medicine, stem cell transplantation represents a promising new approach for restoring damaged tissue and treating a variety of diseases and organ injuries [1]. In cardiac cell therapy protocols, the regeneration of lost cardiomyocytes together with the neovascularization can improve the reactivation of the lost cardiac function and prevent further scar tissue formation [2]. The ultimate goal is to restore both types of damaged tissue, therefore, it is imperative to improve and refine strategy approaches to obtain both cardiac and angiogenic stem cell differentiation contemporarily. Human mesenchymal stem cells (hMSCs), involved in normal tissue renewal and in physiological responses to injuries [3], have been extensively studied for applications in cell therapies [4]. They are able to differentiate into various types of cell lineages, including osteoblasts, chondroblasts, adipocytes, neurons, skeletal and cardiac myoblasts [5]. When these cells are transplanted into an injured myocardium, they can improve and restore the cardiac function [6] and their engraftment efficiency is enhanced when they are “in vitro” committed towards the cardio-angiogenic lineage [7].

The hMSCs, originally isolated from bone marrow, were later obtained from different sources, among which amniotic fluid and the amniotic membrane (AM) from placenta. Placenta is an extra-embryonal tissue and a rich source of stem cells, the use of which can avoid ethical issues [8]. These features render hMSCs, isolated from placenta, an appropriate candidate for applications in cell therapy and regenerative medicine protocols [9].

In this context, the combination of stem cell types and the use of differentiating stimuli (chemical or physical) are important for obtaining the best results [10,11,12].

Different types of chemical agents can be used according to the target tissue: bone morphogenetic proteins (BMPs) in the case of osteogenic differentiation, 5-Azacytidine for cardiac differentiation and the vascular endothelial growth factor (VEGF) for angiogenic differentiation [13,14,15]. Another important cell differentiating agent is Nitric Oxide (NO), a gas produced by various cells within the lower respiratory tract, including inflammatory and epithelial cells. It is able to induce several differentiation processes, such as the cardiac commitment [16,17] of different types of cells, including human mesenchymal stem cells [17,18,19]. It induces an increase of spontaneously contracting cell clusters and also of the cardiac myosin light chain protein expression in treated embryonic stem cells [17].

Physical signals can also be considered an important means for this scope. In particular, Extremely Low Frequency Electromagnetic Field (ELF-EMF) exposure, due to its capability to stimulate and assist osteogenesis, is one of the most commonly used treatments in bone fracture healing. Promising results have also been obtained using ELF-EMF exposure for cell and stem cell differentiation, alone or in combination with a chemical differentiating agent [20,21,22,23,24]. Exposure to physical ELF-EMF stimulus can induce a variety of biochemical processes and changes in gene expression, leading to effects on cellular behavior [25,26]. It alters cell growth and proliferation rate [27], affects synthesis of both mRNA and proteins [21,25,27,28,29,30,31,32,33] and modifies intracellular processes, such as membrane reorganization and ion redistribution [34]. In addition, changes in the cell surface and the induction of nerve regeneration have also been observed [35].

Mechanisms by which electromagnetic fields (EMF) induce functional modifications are still not fully understood. One of the reasons is the lack of reproducibility of results due to the difficulty of having a precise EMF characterization worldwide. To avoid this problem, in our experiments we used an exposure system inside a µ-metal-shielded room able to generate accurate, controlled and reproducible EMF conditions at all times.

Considering this as a starting point and based on our specific experience with non-ionizing EMF radiation [21,22,23,27,28], in this study we isolated and characterized placenta-derived human Amniotic Mesenchymal Stromal Cells (hAMSCs) and investigated the feasibility of obtaining their cardiac and angiogenic commitment using a physical ELF-EMF stimulus and comparing it to the chemical treatment (Nitric Oxide) used as positive control.

## 2. Results

### 2.1. Cell Growth Study of Isolated hAMSCs

**Figure 1:**
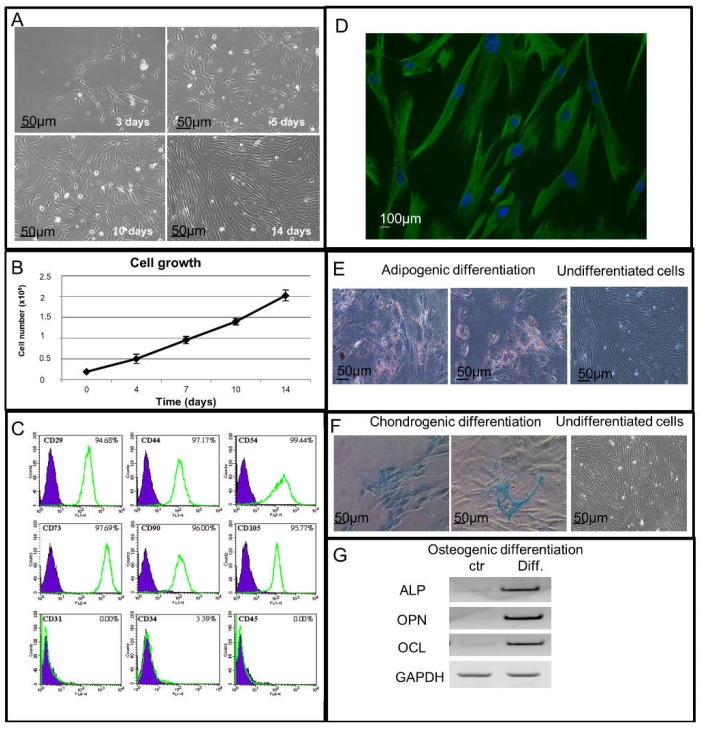
Isolated hAMSC characterization: (**A**) hAMSCs grown with the typical fibroblast like morphology at 3, 5, 10 and 14 days, phase-contrast microscope analysis (20× objective) (*n* = 3); (**B**) time course of hAMSCs’ growth at 4, 7, 10 and 14 days, trypan blue cell exclusion method, data are shown as mean ± SD (*n* = 3); (**C**) hAMSCs’ immunophenotypical characterization for mesenchymal and hematopoietic markers, FACS analysis (*n* = 3); (**D**) hAMSCs’ vimentin expression (green), indirect immunofluorescence analysis. Nuclei are counterstained with Hoechst (blue) (40× objective) (*n* = 3); (**E**) adipogenic differentiation potential of hAMSCs, oil red O staining test (*n* = 3); (**F**) chondrogenic differentiation potential of hAMSCs. Alcian Blue staining test (*n* = 3); (**G**) osteogenic differentiation potential of hAMSCs, Reverse Transcription-Polymerase Chain Reaction (RT-PCR) analysis (*n* = 3).

The placenta-derived hAMSCs were able to adhere to the tissue-culture vessels after 3 days from the isolation. They grew and reached confluence in 14 days acquiring a typical fibroblast-like cell morphology (Figure 1A) and also showed the capability of an exponential growth when seeded on plastic Petri dishes at day 4, 7, 10 and 14 of culture (Figure 1B).

### 2.2. Immunophenotypical and Immunofluorescence Characterization of Isolated hAMSCs

To evaluate the expression of mesenchymal and hematopoietic markers, hAMSCs were analyzed by FACS (Fluorescent Activated Cell Sorting) Cytometer analysis (Figure 1C). The immunophenotypical characterization revealed the expression of mesenchymal Cluster of Differentiation (CD) such as CD73 (97.69%), CD105 (95.77%), CD29 (94.68%), CD44 (97.17%), CD54 (99.44%), CD90 (96%) and the absence of the expression of hematopoietic Cluster of Differentiation (CD) such as CD31, CD34 and CD45 (Figure 1C).

Vimentin, a ubiquitous intermediate filament protein expressed in a wide variety of Mesenchymal Stem Cells types was also studied by indirect immunofluorescence analysis. As reported in Figure 1D, the vimentin expression was highlighted in all the placenta-derived hAMSCs.

### 2.3. Adipogenic, Chondrogenic and Osteogenic Potential Differentiation Study of Isolated hAMSCs

In order to test the hAMSCs capability of differentiating into osteoblast, adipocyte and chondroblast cell lineages, we used specific functional differentiation assays as described in Materials and Methods. By the oil red O staining test, after culturing the cells in adipogenic medium, we observed the presence of red fat storages inside the single multivacuolar cells, typical of the adipogenic differentiation (Figure 1E). When stained with Alcian Blue, the hAMSCs, grown in chondrogenic medium, showed chondrogenic differentiation with blue collagen fibers in their cytoplasm, absent instead in the undifferentiated cells (Figure 1F). By Reverse Transcription-Polymerase Chain Reaction (RT-PCR) analysis, in hAMSCs grown in osteogenic medium, we also demonstrated the osteogenic differentiation capability, highlighted through the expression of osteopontin (OPN), osteocalcin (OCL) and alkaline phosphatase (ALP). All these three osteoblast differentiation markers resulted upregulated in these cells when compared to the control ones (Figure 1G).

### 2.4. Metabolic Activity and Cell Proliferation Study of hAMSCs

**Figure 2:**
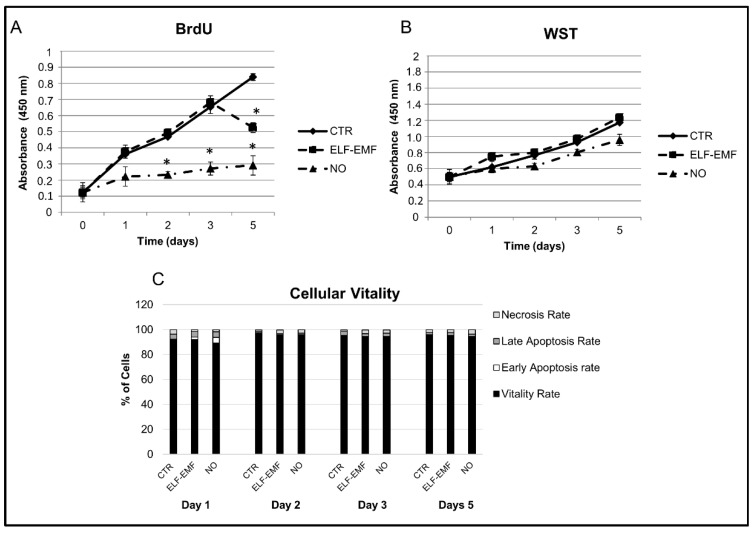
hAMSCs’ metabolic activity (WST assay), cell proliferation (BrdU incorporation assay) and cellular vitality study: (**A**) cell proliferation analysis in hAMSCs control sample (CTR), in 5 days 7 Hz, 2.5 µT exposed cells (ELF-EMF) and in 5 days 0.4 mM Nitric Oxide (NO) treated cells; (**B**) metabolic activity analysis in hAMSCs control sample (CTR), in 5 days 7 Hz, 2.5 µT exposed cells (ELF-EMF) and in 5 days 0.4 mM of Nitric Oxide (NO) treated cells; (**C**) hAMSCs’ vitality and apoptosis study by FACS Cytometer analysis in control cells (CTR), 7 Hz, 2.5 µT exposed cells (ELF-EMF) and in 0.4 mM Nitric Oxide (NO) treated cells at day 1, 2, 3, and 5 of culture. Statistical evaluation of the data was assessed by using a Student’s *t*-test with *p* < 0.05 as the minimum level of significance. Data are shown as mean ± SD. Asterisks identify statistical significance compared to the CTR sample (*p* < 0.05) (*n* = 3).

After studying the mesenchymal and hematopoietic markers’ expression and their capability to differentiate into osteoblast, adipocyte and chondroblast cell lineages, the placenta-derived hAMSCs were exposed for 5 days to physical ELF-EMF stimulus or treated with chemical Nitric Oxide. The effects of the physical agent compared to the chemical one were investigated studying the cells’ metabolic activity and proliferation capability (Figure 2). In the physically exposed hAMSCs, we highlighted a statistically significant decrease in the cell proliferation rate at a later time, from day 4 to day 5, whereas the chemically NO treated cells showed a statistical significant decrease of their proliferation rate at an earlier time (Figure 2A). No difference in metabolic activity was found in the physically exposed cells compared to both the chemically treated cells and the control ones (Figure 2B).

### 2.5. Cellular Vitality and Apoptosis Study in hAMSCs

Apoptotic and viable hAMSCs were studied by FACS analysis using AnnexinV-Fluorescein Isothiocyanate (FITC) and Propidium Iodide labeling. The apoptotic process was studied for control, chemically NO-treated and physically ELF-EMF exposed cells at day 1, 2, 3, and 5 of culture (Figure 2C). The percentage of viable cells in the control and treated samples at each time point were recorded and compared. The analysis performed did not show any change in the hAMSC cell vitality (≥90%) and apoptotic rate (≤4%) in all the samples.

### 2.6. Study of Cardio-Angiogenic mRNA Expression in Chemically and Physically Treated hAMSCs

**Figure 3:**
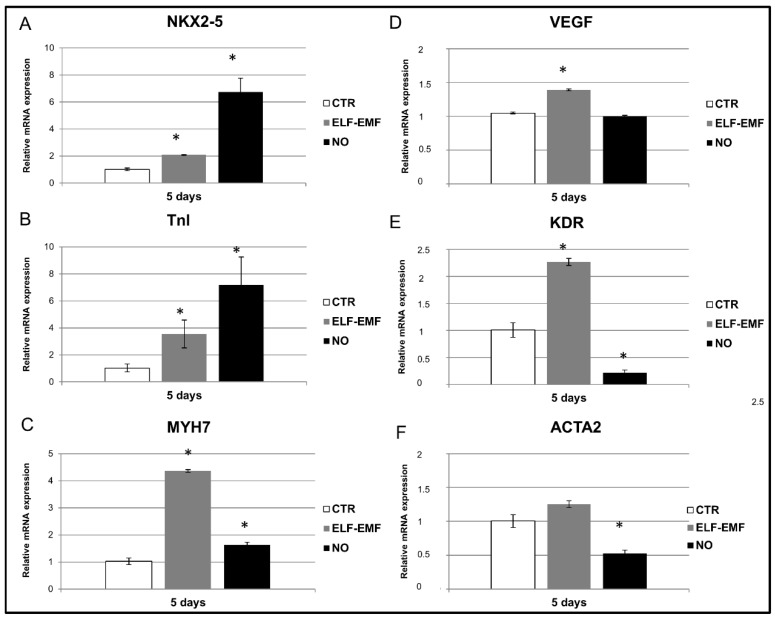
Cardio-angiogenic commitment of hAMSCs by qPCR analysis: (**A**–**C**) study of the NKX2-5, TnI and MYH7 cardiac markers’ expression in 5 days 7 Hz, 2.5 µT exposed cells (ELF-EMF) and in 0.4 mM Nitric Oxide (NO) treated cells compared to control ones (CTR); (**D**–**F**) qPCR analysis of the angiogenic markers VEGF, KDR and ACTA2 in 5 days 7 Hz, 2.5 µT exposed cells (ELF-EMF and in 0.4 mM Nitric Oxide (NO) treated cells compared to control ones (CTR). Statistical evaluation of the data was assessed by using a Student’s *t*-test with *p* < 0.05 as the minimum level of significance. Data are shown as mean ± Standard Deviation (SD). Asterisks identify statistical significance (*p* < 0.05) (*n* = 3).

The cardiac and the angiogenic commitment of hAMSCs exposed to a physical ELF-EMF stimulus or treated with a chemical NO stimulus (for 5 days) were investigated by qPCR mRNA analysis (Figure 3). Exposed hAMSCs showed a statistically significant increase of both cardiac and angiogenic mRNA expressions compared to control cells. In particular, the qPCR analysis revealed a statistically significant upregulation of *NKX2-5* (2-fold), *TnI* (3.5-fold) and *MYH7* (4.4-fold) together with an important increase of mRNA levels for the angiogenic gene *KDR* (2.1-fold) and finally, a small but still statistically significant increase of *VEGF* (1.4-fold), and *ACTA2* (1.25-fold) (Figure 3). Instead, chemically treated hAMSCs showed a statistically significant increase of the cardiac differentiation markers such as *NKX2-5* (6.7 fold), *TnI* (7.2-fold) and *MYH7* (1.6-fold) and a statistically significant decrease of the angiogenic genes such as *VEGF, KDR* (5-fold) and *ACTA2* (1.9-fold) compared to control cells.

### 2.7. Study of Cardio-Angiogenic Protein Expression in Chemically and Physically Treated hAMSCs

**Figure 4:**
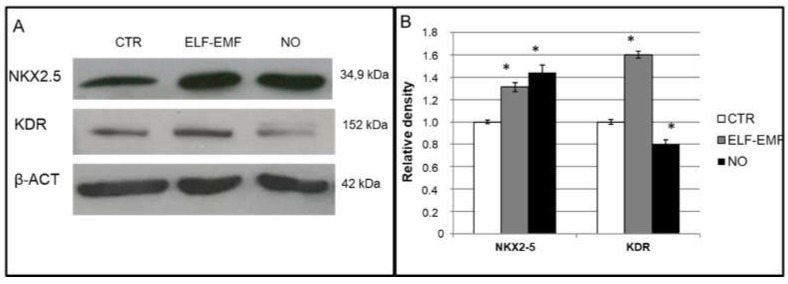
Cardio-angiogenic commitment of hAMSCs by Western Blot analysis: (**A**) study of the protein expression of control cells (CTR), of 5 days 7 Hz, 2.5 µT exposed cells (ELF-EMF) and cells treated with 0.4 mM of Nitric Oxide (NO); (**B**) semi-quantitative analysis by VersaDoc using Quantity One software. Data are shown as mean ± SD. Asterisks identify statistical significance referring to the control sample (*p* < 0.05) (*n* = 3).

**Figure 5:**
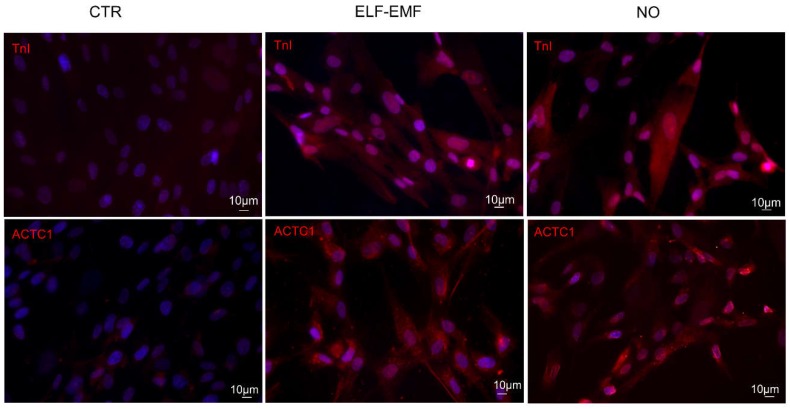
hAMSC protein expression by indirect immunofluorescence analysis of TnI, and ACTC1 (red): control cells (CTR), 5 days 7 Hz, 2.5 µT exposed cells (ELF-EMF) and cells treated with 0.4 mM of Nitric Oxide (NO). Nuclei are counterstained with Hoechst (blue) (*n* = 3).

**Figure 6:**
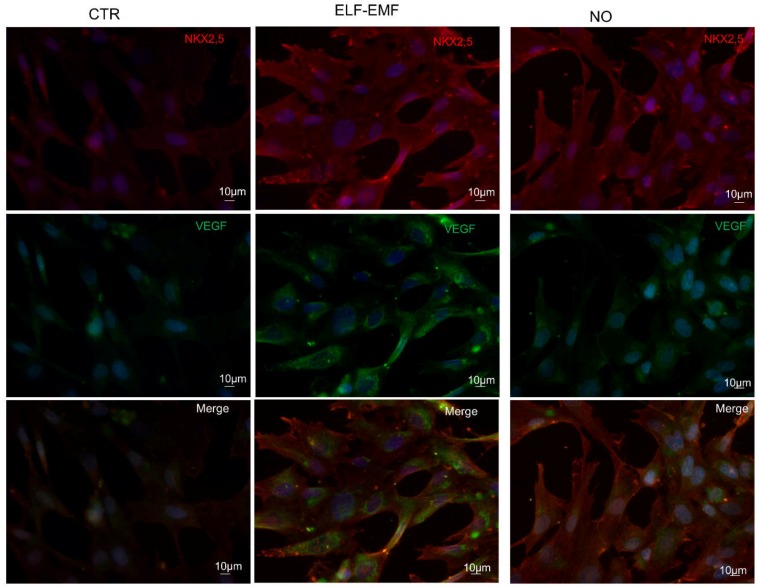
hAMSC protein expression by double indirect immunofluorescence analysis of NKX2-5 (red) and VEGF (green): control cells (CTR), 5 days 7 Hz, 2.5 µT exposed cells (ELF-EMF) and cells treated with 0.4 mM of Nitric Oxide (NO). Nuclei are counterstained with Hoechst (blue) (*n* = 3).

**Figure 7:**
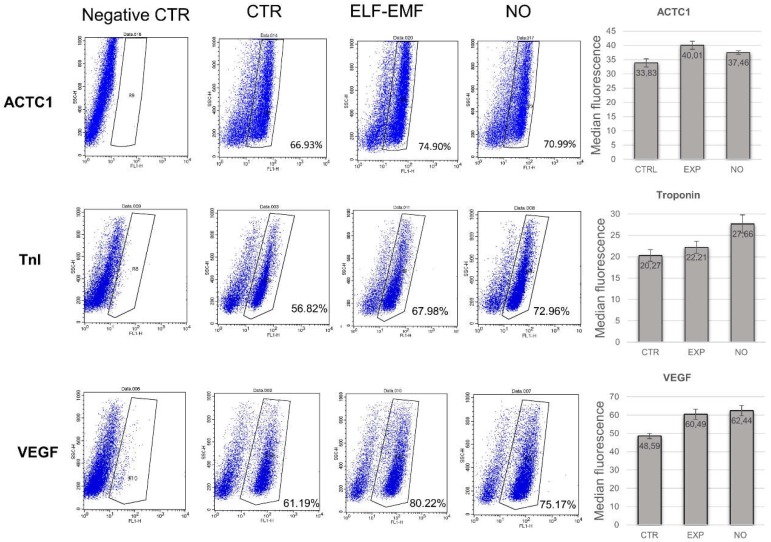
FACS analysis of hAMSCs. The protein expression of TnI, ACTC1, and VEGF after chemical or physical ELF-EMF treatment was evaluated by flow cytometry. Dot plots show the percentage of positive cells: control cells (CTR), physical exposed cells (ELF-EMF) and Nitric Oxide treated cells (NO) (*n* = 3).

The expression of cardiac and angiogenic differentiation markers was examined at translational level by immunofluorescence, western blot and FACS analysis (Figure 4, Figure 5, Figure 6 and Figure 7). Five day ELF-EMF exposed cells showed a statistically significant upregulation of both the cardiac and the angiogenic differentiation protein markers. We also observed that the NO treated hAMSCs showed a statistically significant increase of the cardiac protein marker *NKX2-5* but a statistically significant decrease of the angiogenic protein *KDR* (Figure 4).

The cardiac commitment was further investigated and confirmed by studying the expression of two important cardiac markers such as TnI and Alpha, Cardiac Muscle (ACTC1) by immunofluorescence analysis. Both physically and chemically treated cells showed a significant increase of these markers compared to control ones (Figure 5).

A double immunofluorescence analysis of cardiac and angiogenic markers was also performed in order to evaluate the co-expression of *NKX2-5* and *VEGF* in control, 5 days ELF-EMF and NO treated hAMSCs. The NO treatment showed a greater expression of only the *NKX2-5* marker, whereas in the exposed hAMSCs we observed an increase of the *NKX2-5* and the *VEGF* expression contemporarily (Figure 6). FACS analysis, which was performed to assess the cardiac and angiogenic commitment efficiency on these cells either exposed to ELF-EMF or treated with NO, confirmed an increase of the cardiac and angiogenic protein expressions of TnI, ACTC1 and VEGF compared to control cells (Figure 7).

## 3. Discussion

Bone marrow and adipose tissue are the traditional and main source of human mesenchymal stromal cells for clinical application uses. However, their employment is limited not only because the procedures to isolate hMSCs are highly invasive but also because the proliferating and differentiating potential decreases as the donor’s age increases [36]. Therefore, the identification of an alternative source of hMSCs is an important and a necessary issue to face, and for this scope, a good and promising choice could be the use of neonatal tissues, including placenta and the umbilical cord. The scientific interest for this tissue is generated not only by the potential stem cells that can be isolated from it [37], but also by their intrinsic low immunogenic and immunomodulatory properties [8]. The amniotic membrane (AM) encloses two types of stem cells, epithelial and mesenchymal ones which have different embryological origins. The human Amniotic Epithelial Cells (hAECs), derived from the embryonic ectoderm, form a continuous monolayer in contact with the amniotic fluid. The hAMSCs, deriving from the embryonic mesoderm, are instead spread in the stromal layer underlying the amniotic epithelium. The Mesenchymal and Tissue Stem Cell Committee of the International Society for Cellular Therapy proposed minimal and univocal criteria [38] to better define the isolation, expansion and characterization of hMSCs. They must have the ability to adhere to plastic in standard culture conditions, express CD105, CD73, CD29 and CD90 and lack the expression of CD45 and CD31 surface molecules, and finally they must have osteoblast, adipocyte and chondroblast differentiating potential after in vitro chemical treatments.

In this study, we isolated hAMSCs from placenta, tested and verified them for the requirements of the International Society for Cellular Therapy. After performing the mesenchymal characterization on isolated hAMSCs, we either chemically NO treated them or exposed them to the physical ELF-EMF stimulus and studied the cardiac and angiogenic commitment effects induced on the cells.

We show that 5 day exposed hAMSCs have the same exponential metabolic activity as the control and NO treated cells without a significant difference in cell vitality (Figure 2B). On the contrary, exposed cells show a statistical significant decrease on cell proliferation at a later time compared to NO treated ones in which this effect occurs at an earlier time (Figure 2A).

In order to test if the decrease in cell proliferation is linked to the differentiation process, the transcriptional profile of these cells was studied. As we had expected, in 5 days exposed hAMSCs, we observed an increase of the mRNA expression for both cardiac and angiogenic differentiation markers compared to the chemical NO stimulus in which only an increase of the cardiac markers was found (Figure 3). These results were also confirmed at the translational level by western blot, immunofluorescence and FACS analysis (Figure 4, Figure 5, Figure 6 and Figure 7), demonstrating that the physical ELF-EMF stimulus is able to induce an upregulation of both markers’ expression triggering the two types of commitments contemporarily. The commitment occurs at the same time in the same cells into mostly the cytosol compartment, as highlighted by the immunofluorescence co-localization study (Figure 6)

In our previous studies, we demonstrated that ELF-EMF exposure acts on the cell and stem cell differentiation processes in an irreversible way [22,23,28,29,39,40] through L-type Ca^2+^ channels [21,41], modifying the intracellular processes, and the Ca^2+^ ions redistribution [21,34,41].

A number of mechanisms have been postulated to explain the observed effects through which the physical ELF-EMF stimulus induces functional modifications in the biological system. They have been studied to explain the biological effect of EMF exposure, tuned at calcium ion cyclotron frequency (Ca^2+^-ICR) and some of these theories have appealed more to biochemists [42] while others more to biophysics [43,44,45]. In particular, in 1985, Liboff considered the transport of ions in membrane-bound channels under the action of specific cyclotron frequency, suggesting a mechanism similar to the helical motion of charged particles in free space under the influence of the Lorentz force [44]. Instead, the Lednev theory [42] reports that during the exposure to EMFs, the reorientation of the membrane phospholipids could deform the embedded ion channels altering their dynamics. He considered an ion in its protein-binding site as a dipole and when the energy from the Ion Cyclotron Frequency (ICR) exposure is transferred to the dipole the ion is released in the solution. The most recent approach to the ICR mechanism question has been advanced by Del Giudice et al. [45] who has framed the problem in terms of quantum electrodynamics.

In this study, although we did not investigate the mechanisms involved, on one side, the reported results improve the knowledge on the biological effect of EMF exposure and on the other, they reveal, for the first time, that the exposure to the physical ELF-EMF stimulus is able to induce both cardiac and angiogenic commitment contemporarily in hAMSCs.

These results may have important implications in stem cell therapy approaches as these cells could aid the regeneration of lost cardiomyocytes together with the neovascularization and the reactivation of the lost cardiac function.

Moreover, our results, promising as they are, support the importance of the physical stem cell commitment protocol as a very new and promising tool for obtaining cell commitment with minimal cell manipulation and without any genetic and chemical treatment. It could be used in future regenerative medicine applications to improve ischemic heart repair and in several other diseases for which no treatment is currently available, suggesting that it could be transferred to the patient’s bedside.

## 4. Materials and Methods

### 4.1. Isolation and Culture of hAMSCs

The study was conducted in accordance with the Declaration of Helsinki, and following the protocol approved by the Ethical Committee of the FBF S. Peter Hospital (committee’s reference number *n*. 64/2012/C.B, 27 June 2012). hAMSCs were obtained from human term placenta from healthy women with written informed consent, in general, the cells were processed immediately, then cultured and studied from passages P0 up to P4. The hAMSC isolation, reported in Lisi et al. [19], was generally performed with term placenta dissected from the deflected part of the fetal membranes to minimize the presence of maternal cells. Briefly, homogenous hAMSC populations were obtained by a two-step procedure. Pieces of amniotic membrane were minced and treated for 1 h with a 0.25% trypsin- Acido Etilendiamminotetraacetico (EDTA) solution to remove human amniotic epithelial cells (hAEC), then the supernatant was discarded and the remaining mesenchymal cells underwent a second digestion with 0.1% collagenase IV (Sigma-Aldrich, St. Louis, MO, USA) and 20 µg/mL DNAse I (Sigma-Aldrich) solution in Dulbecco’s modified Eagle’s medium (DMEM) for 2 h. The supernatant was transferred to tubes, neutralized with Fetal Bovine Serum (FBS) then spun at 1200 rpm for 10 min. Each pellet was suspended in complete culture medium containing: DMEM, 10% FBS, penicillin (100 U/mL) and streptomycin (100 μg/mL) (PBI International, Milan, Italy), EGF (10 ng/mL, ImmunoTools, Friesoythe, Germany) and β-mercaptoetanolo (55 µM, Sigma-Aldrich). The amount of hAMSCs from the amniotic membrane is usually about 1 million per gram of tissue. The hAMSCs were cultured on plastic Petri dishes, and maintained at 37 °C in a humid atmosphere containing 5% CO_2_.

### 4.2. Exposure System Description and Characterization

The equipment for ELF-EMF production (solenoid) is set up in a µ-metal-shielded room located in our laboratories [21,27]. This apparatus includes a cellular incubator made of polymethylmethacrylate, a diamagnetic material, where temperature (37 ± 0.1 °C), atmosphere composition (5% CO_2_), and humidity regulation are continuously provided, controlled and recorded by a lab-view program (control system). The main body of the home-produced solenoid is a 5-mm thick polyvinyl chloride cylinder with a diameter of 33 cm, and a length of 3.3 m. It is made of 3300 turns of 1-mm diameter copper wire. It is driven by three amplifiers and a signal generator that creates static and alternate current for the Electromagnetic Field (EMF) production and is able to produce a 0.01 Hz to 1 kHz frequency and 10 nT to 1 mT magnetic flux density. The solenoid has a length/diameter ratio of 10:1, sufficient to ensure extremely high uniformity in the middle. It is used to generate both the static as well as the parallel time-varying fields through the expression: B = µ_0_IN/L, where B is the magnetic flux density, µ_0_ is the magnetic permeability of the free space, I the current, and N/L the ratio of total turns to the length.

4.3. hAMSC Exposure to Physical ELF-EMF Stimulus and Chemical NO Treatment

For every experiment, the protocol treatments used were the following: the cells were grown for 5 days in the following three different conditions: (1) control cells; (2) exposed to physically ELF-EMF stimulus cells; and (3) chemically treated Nitric Oxide cells.

For the EMF treatment, the exposure parameters used were calculated based on the following Lorentz equation:

$$f=\frac{q\left|B_{DC}\right|}{2\pi m}$$

where *q* and *m* are respectively, the ion’s charge and mass; │**B_DC_**│ is the flux density of the applied static Magnetic Field (MF); and *f* is the frequency of the superimposed Electromagnetic Field [44]. In our study, hAMSCs were continuously exposed up to 5 days to a static MF (10 µT) and an alternating ELF-EMF (2.5 µT RMS of intensity) at 7 Hz, matching the cyclotron frequency corresponding to the charge/mass ratio of calcium ion (Ca^2+^-ICR). We applied a very low ELF-EMF intensity, in the range of µT, since it is hypothesized that at this resonance condition, a maximum energy transfer occurs, enabling us to see a biological effect. Under these conditions, the amount of heating due to the Joule effect was negligible, so all the effects reported after cell exposure are related to the Ca^2+^-ICR frequency [22].

For the chemical treatment, the cells were cultured with Nitric Oxide by exogenous supplementation of *S*-nitroso-*N*-acetylpenicillamine (*SNAP*, 0.4 mM, Invitrogen, Carlsbad, CA, USA) at the starting time of the experiment, re-supplemented on day 2 and maintained until day 5 [17].

### 4.4. Phase-Contrast and Immunofluorescence Analysis

The hAMSCs were seeded at a density of 1 × 10^4^ cells/cm^2^ on glass cover slips and grown for 3 days, then fixed in paraformaldehyde 4% at 4 °C for 10 min, washed twice in Ca^2+^/Mg^2+^-free Phosphate Buffered Saline (PBS) and permeabilized at room temperature for 15 min (0.1% Triton X-100, 1% Bovine Serum Albumin (BSA) in PBS; Sigma-Aldrich). The cells were tested by phase-contrast microscope to visualize the cell morphology and by indirect immunofluorescence for detecting the presence of vimentin; NK2 homeobox 5 (*NKX2-5*); cardiac troponin I (*TnI*); Actin, Alpha, Cardiac Muscle *(ACTC1);* and vascular endothelial growth factor (*VEGF*) protein expressions. hAMSCs were incubated with the following primary antibody, in blocking buffer (1% BSA in PBS) for 1 h at room temperature: anti-vimentin (1:200); anti-TnI (1:100), anti-VEGF (1:100) (abcam) anti-ACTC1 (1:100) (Sigma-Aldrich, St. Louis, MO, USA); and anti-NKX2-5(1:100) (abnova). Cells were washed in PBS containing 1% BSA and incubated with an anti-mouse or anti rabbit secondary antibody (1:100) (Chemicon, Billerica, MA, USA) for 1 h at room temperature. Cells were washed in PBS, the nuclei counterstained with Hoechst 33342 (trihydrochloride-trihydrate) and then examined. The images were acquired by an inverted microscope (Olympus IX51, RT Slider SPOT-Diagnostic instruments, Sterling Heights, MI, USA) equipped with 20×, 40× and 60× objectives and with a cooled Fluorescence-Activated Cell Sorting (CCD) camera (Spot RT Slider; Diagnostic Instruments, Sterling Heights, MI, USA). No significant fluorescent signal was detectable with the secondary antibody alone.

### 4.5. hAMSCs Growth

Cells were cultured at density of 1 × 10^4^ cells/cm^2^ in 25 cm^2^ plastic Petri dishes and grown up to 14 days. Cells were harvested with 0.25% trypsin-EDTA (Sigma-Aldrich), washed twice with PBS and viable cells were counted by a Trypan Blue (0.4%) (Sigma-Aldrich) cell exclusion method using a Bürker hemocytometer chamber. Cell counts were performed after 4, 7, 10, and 14 days of culture.

### 4.6. Fluorescence-Activated Cell Sorting (FACS) Analysis

The hAMSCs (1 × 10^4^ cells/cm^2^), were seeded and cultured for three days on plastic Petri dishes. After this, cells were detached with trypsin and washed in cold FACS buffer (2 mM EDTA, 0.5% FBS in PBS 1×), incubated on ice with mouse monoclonal antibodies to human CD105-FITC, CD44-FITC, CD29-FITC, CD34-FITC, CD45-FITC, CD54-PE, CD31-APC (ImmunoTools), CD90-FITC (Milteny, Bergisch Gladbach, Germany) and CD73-PE (BioLegend, San Diego, CA, USA) for 30 min and analyzed by flow cytometry. Isotype control antibodies IgG1-FITC, IgG1, k-PE, IgG1-APC (ImmunoTools) were used for background normalization. Dead cell exclusion was performed adding 5 µg/mL Propidium Iodide to the samples prior to analysis.

For intracellular protein expression analysis the hAMSCs were grown at a cell density of 1 × 10^4^ cells/cm^2^ on petri dishes and cultured up to 5 days. Control, NO-treated and exposed samples were detached with trypsin, washed in cold FACS buffer, fixed in paraformaldehyde 1% at 4 °C for 15 min. and permeabilized for 15 min (1% NP40 in FACS buffer). The cells were incubated in FACS buffer containing 1% BSA for 30 min at 4 °C with the following primary antibodies: anti-TnI-rabbit (1:100), anti-VEGF-rabbit (1:100) (abcam) anti-ACTC1-mouse (1:100) (Sigma-Aldrich). Then the cells were washed in FACS buffer containing 1% BSA and incubated with anti-mouse or anti-rabbit secondary antibodies (1:200) (Chemicon) for 30 min at 4 °C. Anti-mouse or anti-rabbit secondary antibodies were used as negative control.

About 2 × 10^5^ cells were analyzed using a FACSCalibur Cytometer (Becton Dickinson, Franklin Lakes, NJ, USA) and data were processed by Cell Quest Pro software (Becton Dickinson).

### 4.7. hAMSC Adipogenic, Chondrogenic and Osteogenic Differentiation Potential

#### 4.7.1. Adipogenic Differentiation

The hAMSCs were seeded at a density of 1 × 10^4^ cells/cm^2^ and cultured on plastic Petri dishes in DMEM supplemented with 10% FBS, 0.5 mM isobutyl-methyl xanthine (IBMX), 200 μM indomethacin, 10^−6^ M dexamethasone and 10 μg/mL insulin (all Sigma-Aldrich). The cells were cultured for 3 weeks and the medium was replaced every 2–3 days. The cells were fixed in paraformaldehyde 4% and fresh oil red-O solution (Sigma-Aldrich) was used to stain neutral lipids in vacuoles storages in the differentiated cells.

#### 4.7.2. Chondrogenic Differentiation

The hAMSCs were cultured on plastic Petri dishes at a density of 1 × 10^4^ cells/cm^2^ in DMEM low-glucose (Cambrex, East Rutherford, NJ, USA), supplemented with dexamethasone (100 nM), l-ascorbic acid 2-phosphate (50 µg/mL), sodium pyruvate (1 mM), proline (40 µg /mL), ITS (Insulin 5 µg/mL, Transferrin 5 µg/mL, Selenous acid 5 ng/mL; Sigma-Aldrich) and Transforming Growth Factor beta 1(TGF-β1) (5 ng/mL). The cells were cultured for 3 weeks, and the medium was replaced every 2–3 days. The cells were fixed in paraformaldehyde 4% and alcian blue dye was used to stain collagen fibers in the differentiated cells.

#### 4.7.3. Osteogenic Differentiation

The hAMSCs were cultured on plastic Petri dishes at a density of 1 × 10^4^ cells/cm^2^ in DMEM (Sigma-Aldrich) supplemented with 10% FBS, 10 mM β-glycerophosphate, 0.2 mM ascorbic acid, and 10^−8^ M dexamethasone (Sigma-Aldrich). The cells were cultured for 3 weeks, and the medium was replaced every 2–3 days. The RT-PCR analysis was performed to detect the expression of mRNAs for osteopontin (OPN), osteocalcin (OCL) and alkaline phosphatase (ALP) in the differentiated cells.

### 4.8. Cell Metabolic Activity Analysis

The hAMSCs were seeded on three 96-well plates and grown for 5 days. The metabolic activity was detected by a colorimetric assay based on the oxidation of tetrazolium salts (Cell Proliferation Reagent WST-1; Roche Diagnostics, Basel, Switzerland). Water soluble tetrazolium salt (WST-1) reagent, diluted 1:10, was added in the medium after 1, 2, 3, and 5 days of cell culture. After a 2 h incubation in a humid atmosphere, the cells were analyzed by formazan salt formation. The formazan quantification was performed by measuring the absorbance at 450 nm with an ELISA reader (Biotrack II; Amersham Biosciences, Chicago, IL, USA).

### 4.9. Cell Proliferation Analysis

The hAMSCs were seeded on three 96-well plates and grown for 5 days. The hAMSCs proliferation was evaluated by Bromodeoxyuridine incorporation assay (BrdU). 10 mM of Bromodeoxyuridine was added in the cell medium at 1, 2, 3 and 5 days and maintained for 18 h in culture. Cells were then fixed and incubated for 30 min. at room temperature with anti-BrdU antibody (Cell Proliferation Kit; Roche Diagnostics). After incubation with 2,20-Azino-bis (3-ethylbenzothiazoline-6-sulfonic acid) for 30 min, the absorbance of the cell supernatant was measured with an ELISA reader at 450 nm.

### 4.10. Annexin-V Apoptosis Assay

The hAMSCs, were grown at a cell density of 1 × 10^4^ cells/cm^2^ on petri dishes and cultured up to 5 days. Control, NO-treated and exposed samples were harvested at day 1, 2, 3 and 5 and single cell suspensions obtained. According to the manufacturer’s instructions, up to 1 × 10^6^ cells were suspended in 70 µL of Binding Buffer (2.5 mM CaCl_2_ in PBS 1×) and incubated with 5 µL of AnnexinV-FITC (ImmnuTools GmbH, Friesoythe, Germany) for 15 min. at room temperature in the dark. Cells were centrifuged at 250× *g* for 5 min. and then labeled with 5 µg/mL of Propidium Iodide (PI, Sigma-Aldrich). Unlabeled cells were used as negative control. Cells were acquired with a FACSCalibur Flow Cytometer (BDbiosciences, Becton Dickinson, Franklin Lakes, NJ, USA,) equipped with a Cell Quest Software for data analysis.

### 4.11. RT-PCR Analysis

The hAMSCs were seeded at 1 × 10^4^ cells/cm^2^ density on plastic Petri dishes and cultured for 3 weeks in osteogenic medium. Total RNA was extracted from hAMSCs using TRIzol Reagent (Life Technologies, Carlsbad, CA, USA) according to the manufacturer’s instructions. One microgram of total RNA was used to synthesize first-strand cDNA with random primers using 100 U of ImProm-II TM RT-PCR kit (Promega, Madison, WI, USA). The specific primers used for PCR are the following:

ALP, up 5′-ACGTGGCTAAGAATGTCATC-3′; down 5′-CTGGTAGGCGATGTCCTTA-3′; OCL, up 5′-GTGCAGCCTTTGTGTCCAAG-3′; down 5′-TTGGGAGCAGCTGGGATGAT-3′; OPN, up 5′-CCAACGAAAGCCATGACCAC-3′; down 5′-CATGGCTGTGGAATTCACGG-3′; Glyceraldehyde-3-Phosphate Dehydrogenase (GAPDH), up 5′-GTGAAGGTCGGAGTCAACG-3′; down 5′-GGTGAAGACGCCAGTGGACTC-3′; The thermo cycling conditions for each pair of primers were: denaturation at 94 °C for 2 min, followed by amplification at 94 °C for 30 s, annealing for 30 s at 60 °C, elongation for 30 s at 72 °C for 35 cycles.

### 4.12. Real-Time Quantitative RT-PCR Analysis

The hAMSCs were seeded at 1 × 10^4^ cells/cm^2^ density on plastic Petri dishes and cultured for 5 days Total RNA was extracted using TRIzol Reagent (Life Technologies). One microgram of total RNA was used to synthesize first-strand cDNA with random primers using 100 U of ImProm-II TM RT-PCR kit (Promega) according to the manufacturer. The quantification of all gene transcripts was carried out by real-time RT-PCR. The specific primers for cardiac differentiation markers such as NK2 homeobox 5 (NKX2-5, a transcription factor with functions in heart formation and development), cardiac troponin I (TnI, a cardiac regulatory protein controlling the calcium mediated interaction between actin and myosin), myosin, heavy chain 7 (MYH7, correlated with the contractile velocity of the cardiac muscle) are shown in Table 1. The specific primers for angiogenic differentiation markers such as vascular endothelial growth factor (VEGF, an essential angiogenic factor for vascular endothelial cells), kinase insert domain receptor (KDR, a type III receptor tyrosine kinase which is the main mediator of VEGF-induced endothelial proliferation, survival and migration) and actin, alpha 2, smooth muscle, aorta (ACTA2, contributes to the ability of blood vessel muscles to contract, allowing the arteries to maintain their shape) are shown in Table 1. The reaction was also carried out in the absence of reverse transcriptase to check for genomic DNA amplification. Experiments were conducted to contrast relative levels of each transcript and endogenous control GAPDH in every sample. The data were analysed using the equation described by Livak and Schmittgen [46], as follows:

Amount of target—2^−ΔΔ*C*t^.

Δ*C*t = (average target *C*t − average GAPDH *C*t)

ΔΔ*C*t = (average Δ*C*t treated sample − average Δ*C*t untreated sample)

Before using ΔΔ*C*t method for quantification, we performed a validation experiment to demonstrate that efficiency of target genes and reference GAPDH were equal. Real-time PCR was conducted using Sybr Green I Mastermix (Applied Biosystems, Carlsbad, CA, USA) using an ABI PRISMTM 7000 Sequence Detection System. Each reaction was run in triplicate and contained 0.5 µL of cDNA template along with 250 nM primers in a final reaction volume of 25 µL. Cycling parameters were 50 °C for 2 min, then 95 °C for 10 min. to activate DNA polymerase, then 40 cycles of 95 °C for 15 s and finally 60 °C for 1 min. Melting curves were performed using Dissociation Curves software (Applied Biosystems) to ensure that only a single product was amplified. As negative controls, we used tubes where RNA or reverse transcriptase was omitted during the RT reaction.

### 4.13. Western Blotting Analysis

The hAMSCs were seeded at 1 × 10^4^ cells/cm^2^ density on plastic Petri dishes, cultured for 5 days and then the SDS-polyacrylamide gel electrophoresis (SDS-PAGE) was carried out. Equal amounts of proteins from control, exposed and NO treated cells were loaded for each lane. Electrophoresis was carried out in a range between 6% and 12% SDS polyacrilamide gel at 60 V for 2 h. Transfer on nitrocellulose membranes (BioRad, Hercules, CA, USA) was subsequently performed at 300 mA for 2 h. After a 5% fat-free-milk block for 1 h at room temperature, membranes were incubated with the following monoclonal antibodies: anti-NKX2-5 (1:1000), anti-KDR (1:500), anti-β-ACT (1:2000), (and revealed by a chemiluminescence (ECL) system (Amersham Biosciences). Protein expression levels were determined semiquantitatively by a densitometric analysis with Quantity One software-4.4.0 (BioRad) and the control β-ACT protein was used to calculate the relative density.

### 4.14. Statistical Analysis

Each experiment was performed three times (*n* = 3) for phase-contrast, immunofluorescence microscope analysis, FACS analysis and western blot analysis, the results of one representative experiment are shown. The results are presented as mean ± SD. The significance of the difference was evaluated using the Student’s *t* test with *p <* 0.05 as the minimum level of significance.

## Figures and Tables

**Table 1:** Primers used for real-time reverse transcriptase polymerase chain reaction.

Target	Sequence	Annealing Temperature (T)
*VEGF*	5′-CTTGGGTGCATTGGAGCCT-3′ 5′-CTGCGCTGATAGACATCCAT-3′	60 °C
*KDR*	5′-CAGCATCACCAGTAGCCAG-3′ 5′-TTATACAGATCTTCAGGAGCTT-3′	60 °C
*ACTA2*	5′-ATGAAGATCCTGACTGAGCG-3′ 5′ –GCAGTGGCCATCTCATTTTC-3′	60 °C
*NKX2-5*	5′-CAGCGACCCCGACCCAG-3′ 5′-GCTTCCTCCGCCGTCGC-3′	60 °C
*MYH7*	5′-CAGAAGAAGAAGATGGATGC-3′ 5′-CGCTGGTGTCCTGCTCCT-3′	60 °C
*TnI*	5′-GGACAAGGTGGATGAAGAGA-3′ 5′-AGGGTGGGCCGCTTAAACT-3′	60 °C
*GAPDH*	5′-CATCATCTCTGCCCCCTCT-3′ 5′-CAAAGTTGTCATGGATGACCT-3′	60 °C
